# Acute Hypoxic Exposure and Right Ventricular Adaptation: Morphological Remodeling, Functional Dynamics, and Hemodynamic Implications in Healthy Individuals

**DOI:** 10.33549/physiolres.935502

**Published:** 2025-08-01

**Authors:** Tianyuan WANG, Ji MA, Yafeng SONG

**Affiliations:** 1 Qinghai Normal University, Xining, China; 2 Institute of Sport and Health Science, Beijing Sport University, Beijing, China

**Keywords:** Hypoxia, Right ventricle, Pulmonary vascular resistance

## Abstract

From previous studies, the right heart is considered less critical than the left heart in maintaining normal global hemodynamic performance. However, there is now substantial evidence underscoring the importance of comprehensive right ventricular (RV) function. Acute hypoxia is associated with an increase in pulmonary artery pressure (PAP), leading to changes in RV hemodynamics. Moreover, hypoxia may directly affect the RV. The current literature assessing the impact of acute hypoxia on RV hemodynamics remains insufficiently elucidated. This paper aims to delineate the effects of acute hypoxia exposure on the RV in healthy individuals.

## Introduction

The right ventricle (RV) is defined as a thin-walled, crescent-shaped structure, with one aspect linked to systemic venous return and the opposing aspect connected to pulmonary circulation [1]. From a physiological perspective, the primary function of the RV is to channel all venous return into the pulmonary circulation while simultaneously preventing an increase in right atrial pressure (RAP) [2]. Given its thin and crescent-shaped wall configuration, the RV demonstrates compliance and can accommodate and eject a relatively large volume of blood when myocardial shortening is constrained [3], consequently, in contrast to the left ventricle (LV), which functions primarily as a pressure pump, it is better suited for accommodating significant increases in blood volume rather than pressure [4].

As altitude increases, the partial pressure of oxygen in the atmosphere decreases concomitantly with the reduction in atmospheric pressure, leading to a progressive decline in available oxygen [5]. Consequently, exposure to high altitudes results in diminished arterial oxygen content, thereby triggering an adaptive process within the body, this process encompasses a series of physiological responses across various organs and systems aimed at ensuring adequate delivery of oxygen to tissues [6]. The cardiovascular system's response to hypoxia is notably dynamic, as the duration of exposure at high altitudes extends, its adaptation transitions from an acute phase (within the initial hours) to a chronic state [7]. Acute responses are primarily characterized by heightened sympathetic nervous tone, which arises from hypoxia-induced stimulation of peripheral chemoreceptors [5]. Furthermore, the degree of autonomic nervous system activation escalates proportionally with increasing levels of hypoxia and continues to intensify throughout the entirety of altitude exposure [8,9].

In the context of high-altitude diagnosis, heart failure is predominantly right-sided in nature [10]. Research indicates that acute right heart failure may occur in healthy individuals exposed to high altitudes due to excessive overload on the RV pressure [11]. This phenomenon can be attributed to the contraction and remodeling of pulmonary vessels under hypoxic conditions, leading to an increased afterload on the RV, potentially exacerbated by negative inotropic effects resulting from environmental low-pressure hypoxia [12]. The existing literature evaluating the impact of acute high-altitude exposure on RV function remains contentious [13,14]. The reasons for these discrepancies are not well understood and may stem from significant variations in methodologies and study designs across studies, as well as non-compliance with established guidelines for assessing RV function and morphology [15]. A comprehensive understanding of the physiological responses of the RV to acute hypoxia is crucial for elucidating mechanisms underlying RV dysfunction and various cardiopulmonary disorders. Therefore, this article aims to elaborate on how healthy individuals' RV responds to acute hypoxic exposure and altitude ascent.

## The Impact of Acute Hypoxia Exposure on the Morphology of RV

Individuals who have long resided at sea level enter high-altitude environments, the low partial pressure of oxygen activates hypoxic pulmonary vasoconstriction (HPV) [16]. This is a physiological compensatory mechanism that optimizes ventilation-perfusion matching through local vasoconstriction, aiming to alleviate the influence of hypoxia on the organism [17]. However, pulmonary vasoconstriction causes an increase in pulmonary vascular resistance (PVR), raises the afterload of the RV, and forces it to pump blood at a higher pressure, thereby triggering an increase in mean pulmonary artery pressure (mPAP) [18]. The myocytes of the RV sense this increased mechanical load through sarcomeres, which are the key sites for mechanotransduction [19], triggering downstream signaling pathways that regulate cellular gene expression and protein synthesis [20], thus promoting the proliferation of myocardial cells, leading to an increase in their number, widening, and elongation, and ultimately contributing to RV hypertrophy as well as morphological alterations [21]. This adaptive remodeling is a core compensatory mechanism for the RV to maintain the homeostasis of ventricular wall stress.

With increasing altitude, both the dimensions of the RV and the volume of the right atrium progressively enlarge [22]. Netzer [23] investigated changes in RV dimensions during acute normobaric hypoxia exposure and concluded that all subjects exhibited a significant linear increase in RV dimensions with ascending altitude, concurrently accompanied by an elevation in right ventricle systolic pressure (RVSP). However, some studies indicate that there are no alterations in the morphological structure of the RV following simulated acute hypoxia exposure [16,17,24,25]. The discrepancies observed across these experimental outcomes can be attributed to two factors: firstly, variations in hypoxia levels employed during experimentation; secondly, potential brief interruptions of hypoxia occurring during measurement periods. Furthermore, research has demonstrated that merely one minute of adequate oxygenation can nearly halve the impact of hypoxia on RVSP; additionally, healthy young subjects exhibit immediate compensatory responses from their vascular systems against HPV, effectively reversing RV dilation [23].

## The influence of acute hypoxia exposure on RV function

Previous research has indicated that the myocardium might restrict its pumping function *via* the regulatory mechanism of the central nervous system in order to avoid progressive myocardial ischemia induced by insufficient coronary artery oxygen supply, this protective mechanism (termed the "governor" by early scholars) is accomplished by lowering the neural recruitment level of skeletal muscles, rather than being directly driven by myocardial hypoxia, thereby maintaining the balance of myocardial oxygen supply and demand while preventing potential arrhythmias or functional impairments [26,27]. Emerging evidence suggests that hypoxia itself may precipitate cardiac dysfunction as a consequence of prolonged and continuous low oxygen availability; however, this phenomenon appears predominantly under conditions of sustained exposure [28]. It has been established that chronic hypoxia leads to inhibition of both contraction and relaxation functions in the myocardium of both ventricles [29].

Non-invasive evaluations of RV function under acute hypoxic conditions indicate that RV performance is compromised following acute exposure at FiO_2_ = 0.11 [23] and FiO_2_ = 0.125 [30]. The study conducted by Stembridge [31] similarly concluded that short-term hypoxia can inflict varying degrees of impairment on RV function. Nevertheless, not all investigations assessing RV functionality in hypoxic environments have reported consistent findings. For instance, Forbes [32] tested subjects in a supine resting position during the transition from FiO_2_ = 0.21 to FiO_2_ = 0.12 and found that RV function was preserved during acute hypoxia, with enhancements observed in contractility, diastolic function, and energetics. In subsequent research by this author, similar results were obtained through invasive assessments of RV function when FiO_2_ was set at 0.12 in response to acute hypoxia [33]. Furthermore, experimental outcomes from the Operation Everest II have raised questions regarding the prevailing notion that hypoxia inhibits cardiac function, during the period of staying at high altitude, the relationship between RAP, which is used as an alternative indicator of RV performance, and the decrease in stroke volume caused by hypoxia remains unchanged, leading to conclusions about retained RV functionality despite such circumstances [34]. Maufrais [35] conducted an echocardiographic assessment at an altitude of 5,085 meters and did not observe any signs of RV contractile dysfunction, thereby providing further support for the hypothesis that the myocardium demonstrates significant tolerance to high altitude. These discrepancies may be attributed to variations in study design. In Maufrais's study [35], The high-altitude assessment was carried out on the second day when the subjects arrived at an altitude of 5,085 meters; however, in Stembridge's study [31], assessments were conducted ten days post-arrival at high altitude. Consequently, the continuous increase in physiological load under hypoxic conditions may play a critical role in the development of RV dysfunction among individuals arriving at elevated altitudes over several weeks [35].

### Effect on contraction function

A healthy RV has been demonstrated to possess a significant contractile reserve, capable of increasing contractile force by three to four times during the transition from rest to peak exercise, at least under normoxic conditions [36]. As altitude increases, the RV undergoes dilation, and its contractile function is impaired to varying degrees [22]. The decline in myocardial contractility may be attributed to compromised myocardial oxygenation resulting from decreased coronary arterial oxygen tension, diminished coronary blood flow, or both [26].

Research findings regarding the effects of acute hypoxic exposure on RV contractile function are inconsistent. Although some studies have suggested that the increase in afterload resulting from acute hypoxia may enhance RV contractile function [37,38], others have reported no change [17] or even impaired contractility [39–41]. These discrepancies in experimental results within the current literature may stem from substantial differences in research methodologies and study designs. It has been documented that hypoxia exerts a negative inotropic effect on both intact animal models [42] and isolated myocardial fibers [43]. In certain patients sensitive to hypoxia, RV dysfunction is thought to arise from the direct negative inotropic effects of hypoxia on cardiomyocytes as well as reduced levels of oxygenation [44]. A recent investigation has substantiated this direct negative inotropic effect, it demonstrated that enhancing oxygen delivery to the myocardium of lambs subjected to acute hypoxia can preserve RV contractile function [45].

### Effect on diastolic function

High-altitude exposure is consistently recognized as a cardiac stressor, under hypoxic conditions, the diastolic function of both ventricles is typically compromised [46]. Owing to the inherent structural characteristics of the RV, including a thinner myocardial wall and a complex anatomical configuration, its pressure compensation reserve is conspicuously weaker than that of the LV [47]. Under hypoxic circumstances, this structure-function property results in a significantly greater extent of influence on the diastolic function of the RV compared with that of the LV [17].

Previous studies have demonstrated that acute hypoxia can trigger responses in pulmonary vessels, leading to transient pulmonary arterial hypertension and a decline in RV diastolic function [48–51]. Furthermore, research indicates that acute exposure to hypoxic environments reduces the E/A ratio of tricuspid inflow, the E/A ratio is a crucial echocardiographic indicator in the assessment of cardiac diastolic function, reflecting the equilibrium between the active relaxation capacity of the ventricles and their passive filling properties, a decline in the E/A ratio suggests the occurrence of functional abnormalities in the diastole of both ventricles during acute hypoxia [17,52,53]. Upon returning to sea level, the E/A ratio can revert to baseline values [54]. Two potential explanations exist for the changes observed in early RV diastolic function due to hypoxia. Firstly, hypoxia may alter the mechanisms governing calcium uptake by active cells, specifically, hypoxia suppresses the activity of sarcoplasmic reticulum ATPase (Ca^2+^-ATPase), attenuates the efficiency of calcium ion reuptake during diastole, causes a delay in cytoplasmic calcium clearance, and consequently prolongs the myocardial diastolic time [43]. The second explanation relates to contraction-relaxation coupling, it posits that hypoxia induces an extension of the diastolic phase correlated with both the magnitude and timing of contraction load [55].

## The Effects of Acute Hypoxia Exposure on RV Hemodynamics

Hypoxic exposure is associated with significant alterations in cardiovascular function. In the early stage of hypoxia, the cardiovascular responses are manifested as tachycardia, an increase in cardiac output, unchanged stroke volume, and a possibly transient and mild elevation of blood pressure [12]. The initial response of the cardiovascular system to hypoxia can be attributed to the combined effects of systemic hypoxic vasodilation, activation of the sympathetic chemoreflex and reduced blood volume, the mechanism of reduced blood volume is closely related to the compensatory increase in cardiac output, the increase in cardiac output can enhance renal perfusion under hypoxic stimulation, thereby accelerating fluid excretion through diuresis and ultimately leading to a decrease in blood volume [56].

The influence of hypoxia on the cardiovascular system typically exhibits a notable time-delay effect, the core mechanism involves the dynamic reconfiguration of heart rate (HR) and stroke volume, which is accomplished gradually through physiological adaptation over several days to weeks [12]. Research indicates that although acute hypoxia exposure can trigger a compensatory increase in HR, in a chronic hypoxic environment, the cardiovascular system reconstructs the oxygen transport efficiency by reducing the maximum HR and decreasing stroke volume [57,58]. This process is closely related to slow adaptation mechanisms such as elevated blood viscosity and reduced ventricular filling pressure [59]. It is worth noting that even if hypoxemia is promptly corrected through oxygen inhalation, cardiac output still cannot fully recover to the baseline level, suggesting that deep adaptations such as the regulation of myocardial contractility and the remodeling of vascular tone require several weeks and cannot be reversed rapidly through short-term intervention [58]. Most of the cardiovascular alterations resulting from acute high-altitude exposure, such as augmented cardiac output and elevated PAP, are typically reversible within several weeks to months *via* physiological acclimation, specific interventional measures (e.g., pulmonary vasodilators) may partially restore the maximal exercise capacity; however, the primary adaptation mechanism is mediated by the systemic adjustments in response to the hypoxic environment [12].

Acute hypoxia leads to the activation of the chemical reflex of the sympathetic nervous system [60]. In response to the heightened sympathetic nervous activity and the concomitant attenuation of vagal tone, along with a significant increase in HR and cardiac output, blood pressure initially decreases due to hypoxia-induced vasodilation, resulting in reduced vascular resistance, however, as the adaptation process progresses, blood pressure typically rises to levels exceeding those observed at sea level [5]. The magnitude of blood pressure elevation is directly proportional to the altitude achieved and remains consistent throughout the duration of exposure [8,61]. Some evidence suggests that mild systemic hypertension may improve with extended stays at high altitudes, where residents generally exhibit lower prevalence rates of hypertension compared to those living at sea level [62].

### Effect on cardiac output

The enhancement of cardiac output observed during high-altitude exposure is primarily attributed to an elevation in HR, while stroke volume remains unchanged or may decrease [12]. Two to three days after exposure to hypoxia, stroke volume declines, HR remains elevated, and cardiac output gradually returns to a level comparable to or slightly below that at sea level [63,64]. The pattern of variation in cardiac output during acute high-altitude exposure is at least partially linked to the activation of the sympathetic nervous system [17,60]. This phenomenon is evidenced by increased catecholamine levels in plasma and urine [65,66], as confirmed by microscopic neurography records [60,67]. Stenberg's study [68] demonstrated that individuals exposed to acute hypoxia exhibit an increase in cardiac output at any given submaximal workload. Similarly, Forbes's research [33] concluded that a healthy RV can adequately maintain cardiac output under acute hypoxic conditions, particularly during short-term submaximal exercise.

### Effect on stroke volume

Following acute exposure to reduced atmospheric pressure, stroke volume does not decline immediately [68]. In the simulated extremely high-altitude environment, the stroke volume, serving as the dependent variable of ventricular contractility and the filling pressures of the LV and RV, remains stable, this phenomenon verifies that the myocardial contractile efficacy did not undergo significant impairment [59], which has been confirmed by the relationship between peak systolic pressure and end-diastolic volume in the LV [69]. Research indicates that following 2 to 3 days of acclimatization, stroke volume typically decreases [63], this decrease is generally results in diminished left ventricular filling [70–72]. Meanwhile, cardiac output gradually reverts to the baseline level, while HR keeps increasing [12,56]. Two mechanisms have been proposed to explain this reduction in stroke volume. Firstly, the reduction of blood volume is potentially capable of causing a decrease in stroke volume through the lowering of ventricular filling pressure [26,73]. Secondly, long-term pulmonary arterial hypertension will result in myocardial remodeling and a decline in left ventricular compliance, thereby directly reducing stroke volume [52,73]. Even after prolonged residence at high altitudes for several years, stroke volume may still exhibit an indefinite decrease, however, this decrement is reversible upon return to sea level [58]. The reduction in cardiac output resulting from decreased stroke volume significantly contributes to the impairment of human work capacity at high altitudes [74].

## The Interaction Between the Pulmonary Vascular System and RV Under Acute Hypoxic Exposure

Exposure to hypoxia induces contraction and remodeling of pulmonary blood vessels [12,17,75], which is manifested by the elevation of its key physiological indicator – PVR [76]. Under hypoxic conditions, HPV is anticipated to result in remarkable alterations in the function of the RV on account of augmented afterload [34]. The RV exhibits limited adaptability to abrupt changes in afterload, such as those occurring during acute pulmonary thromboembolism [77] or sudden exposure to high-altitude environments. This phenomenon is particularly pronounced in susceptible individuals [11]. Prolonged exposure results in a chronic elevation of RV afterload, with residents of high-altitude regions potentially experiencing marked RV dysfunction or failure [22]. In these scenarios, RV dysfunction may not solely be attributed to elevated afterload; it can also arise from increased ventricular wall stress and the direct negative inotropic effects of hypoxia on cardiomyocytes due to diminished oxygenation [44].

### The impact of elevated PVR on RV

Acute exposure to high altitude induces significant cardiac and hemodynamic alterations, primarily associated with increased PVR due to hypoxia [12,35,52,54]. Elevated PVR levels can lead to an increase in RV end-diastolic pressure, and prolonged exposure may result in RV dilation and hypertrophy [78,79]. Such changes in RV morphology can subsequently reduce RV systolic function, potentially culminating in heart failure [76,80]. Furthermore, the rise in PVR also diminishes maximal cardiac output, thereby impairing exercise performance [14,81]. In healthy individuals, the pulmonary vascular system is capable of making regulatory adjustments to substantial fluctuations in blood flow without considerable changes in pressure, thus, the RV typically does not experience pressure overload [4]. However, there is notable inter-individual variability regarding the response of pulmonary vessels to hypoxia. In certain particularly susceptible individuals, the RV may fail to achieve a stable adaptive state as PVR and PAP gradually escalate over weeks or months, ultimately leading to RV failure. If alveolar hypoxia remains uncorrected during this process, it can become life-threatening [82].

### The PAP level rises following acute hypoxia exposure

Over the past several decades, there has been a notable increase in the number of individuals traveling to high-altitude regions for economic or recreational purposes [83]. For most mammals, including humans, an elevation in PAP is an inevitable consequence during the initial phase of ascent to high altitudes [49]. In the majority of cases, this increment in PAP is moderate, allowing the ventricles to adapt successfully and achieve a stable state that can persist for many years [82]. Research indicates that acute exposure to high altitudes among populations with prolonged residence at sea level can result in mPAP rising to 20 mmHg and 25 mmHg [53], the mPAP observed within general populations at high altitudes is approximately 7 mmHg higher than that recorded at lower elevations [84]. During acute exposure to elevated altitude, increased mPAP primarily results from HPV, which subsequently elevates RV afterload, impairing RV function and leading to ventricular mismatch [85]. Notably, mPAP tends to rise with increasing exercise intensity in high-altitude environments [76,86]. During exercise in high-altitude areas, increases in cardiac output lead to more pronounced elevations in PAP compared to similar activities performed at sea level [87]. Evidence suggests that an increase in mPAP or PVR may, conversely, limit exercise capacity in high-altitude environments [12,88]. In Operation Everest II [34], it was found through direct measurement by right heart catheterization that the mPAP at rest rose from 15 ± 0.9 mmHg at sea level to 34 ± 3.0 mmHg at the simulated altitude of 7,620 meters, and further increased during exercise. Operation Everest III [70] employed non-invasive ultrasound Doppler technology, indicating that the systolic PAP (estimated by the RV/RA pressure gradient) soared from 19.0 ± 2.4 mmHg at sea level to 40.1 ± 3.3 mmHg at 8,000 meters. Both studies have indicated that with the aggravated hypoxia resulting from the increase in altitude, PAP keeps rising continuously, and attributed this hypoxia-induced secondary pulmonary hypertension to the increase in PVR, the mechanisms of which include hypoxic vasoconstriction and potential vascular remodeling[34,70]. Clinically, pulmonary arterial hyper-tension is defined as mPAP exceeding 20 mmHg at rest or greater than 30 mmHg during exertion [89]. High-altitude medical experts have even employed a higher threshold, namely, greater than 30 mmHg [90]. Furthermore, it has been verified that pulmonary arterial hypertension is related to RV systolic dyssynchrony, RV systolic dyssynchrony is jointly driven by the increased mechanical afterload caused by elevated PAP, electrical heterogeneity, and metabolic disorders induced by systemic hypoxia, and has been recognized as a crucial characteristic of pulmonary hypertension [24]. Indeed, the adaptation mechanism of the RV to pulmonary hypertension is essentially homeometric adaptation, namely, by enhancing myocardial contractility to increase the end-systolic elasticity of the ventricle, maintaining the dynamic matching of the elastic characteristics between the ventricle and the arterial system, thereby maintaining ventricular-arterial coupling and ensuring the relative stability of cardiac output and energy efficiency (the blood flow output per unit energy consumption) [91]. Notably, following prolonged residence back at sea level, instances of pulmonary arterial hypertension can be reversed [76].

### HPV under acute hypoxic exposure

Acute hypoxia induces acute vasoconstriction in the pulmonary circulation [92]. Although acute hypoxic vasoconstriction acts as a protective mechanism for redistributing blood flow to well-ventilated lung areas and thereby enhancing ventilation-perfusion matching, chronic hypoxia can induce pathological remodeling of the pulmonary vessels, which is characterized by smooth muscle proliferation, intimal fibrosis, and irreversible increase of PVR, eventually resulting in pulmonary arterial hypertension [4]. Research indicates that acute HPV [93] and the activation of the sympathetic nervous system [94] might constitute significant mechanisms contributing to the initial elevation of PAP in individuals when exposed to hypoxic conditions at high altitudes. Acute HPV is considered an adaptive response of the pulmonary circulation to localized alveolar hypoxia, optimizing ventilation-perfusion matching and gas exchange by redistributing blood flow from poorly ventilated lung segments to those with optimal ventilation [75]. The mechanisms linking HPV and pulmonary arterial hypertension in acute and chronic altitude maladaptation syndromes involve multi-level interactions. Acute hypoxia induces calcium influx and vasoconstriction by inhibiting potassium channels in pulmonary vascular smooth muscle, while long-term exposure activates the hypoxia-inducible factor pathway, which promotes vascular smooth muscle proliferation and intimal fibrosis, thereby leading to an increase in PVR [22]. However, the molecular regulatory network of HPV, including the roles of reactive oxygen species and microRNAs, as well as the causal relationship between oxidative-inflammatory-nitrosative stress and vascular remodeling, remain to be fully elucidated [22,95,96]. Furthermore, significant interindividual variability exists regarding acute HPV responses among humans [97]. For instance, RV dysfunction associated with HPV is notably prevalent during exacerbations of respiratory conditions such as chronic obstructive pulmonary disease and diffuse fibrotic lung diseases [98]. Additionally, episodes of transient hypoxemia are often accompanied by sleep-related breathing disorders, with obstructive sleep apnea being the most common type observed [6]. HPV also contributes to a transient increase in PAP during sleep in patients suffering from obstructive sleep apnea [99]. In summary, the acute hemodynamic burden imposed by HPV on RV function is frequently observed during exacerbations of various cardiopulmonary disorders, thus, understanding its pathophysiology may aid in developing more effective treatment strategies.

## Conclusion

Acute hypoxia is commonly associated with mild pulmonary arterial hypertension, increased HR and cardiac output, stable stroke volume, and alterations in RV function. These changes can be attributed to elevated PAP, activation of the sympathetic nervous system, isobaric adaptation of RV function to afterload, and the cumulative effects of hypovolemia. In conclusion, cardiac function is generally well preserved under acute hypoxic conditions. Investigating the physiological principles underlying the RV's response to hypoxia is crucial for a comprehensive understanding of the mechanisms driving acute RV dysfunction and certain diseases prevalent in low-altitude regions.

## References

[1] Sanz J, Sánchez-Quintana D, Bossone E, Bogaard HJ, Naeije R (2019). Anatomy,function, and dysfunction of the right ventricle: JACC state-of-the-art review. J Am Coll Cardiol.

[2] Pinsky MR (2016). The right ventricle: interaction with the pulmonary circulation. Crit Care.

[3] Rigolin VH, Robiolio PA, Wilson JS, Harrison JK, Bashore TM (1995). The forgotten chamber: the importance of the right ventricle. Cathet Cardiovasc Diagn.

[4] Budev MM, Arroliga AC, Wiedemann HP, Matthay RA (2003). Cor pulmonale: an overview. Semin Respir Crit Care Med.

[5] Cornwell WK, Baggish AL, Bhatta YKD, Brosnan MJ, Dehnert C, Guseh JS, Hammer D, Levine BD, Parati G, Wolfel EE (2021). Clinical implications for exercise at altitude among individuals with cardiovascular disease: a scientific statement from the american heart association. J Am Heart Assoc.

[6] Mamazhakypov A, Sartmyrzaeva M, Kushubakova N, Duishobaev M, Maripov A, Sydykov A, Sarybaev A (2021). Right ventricular response to acute hypoxia exposure: a systematic review. Front Physiol.

[7] Baggish AL, Wolfel EE, Levine BD, Swenson ER, Bärtsch P (2014). Cardiovascular System. High Altitude.

[8] Levine BD (2015). Going high with heart disease: the effect of high altitude exposure in older individuals and patients with coronary artery disease. High Alt Med Biol.

[9] Bärtsch P, Gibbs JS (2007). Effect of altitude on the heart and the lungs. Circulation.

[10] Naeije R (2013). RV is doing well at high altitudes!--always?. JACC Cardiovasc Imaging.

[11] Huez S, Faoro V, Vachiéry JL, Unger P, Martinot JB, Naeije R (2007). Images in cardiovascular medicine. High-altitude-induced right-heart failure. Circulation.

[12] Naeije R (2010). Physiological adaptation of the cardiovascular system to high altitude. Prog Cardiovasc Dis.

[13] Richalet J-P, Pichon A, Gaine SP, Naeije R, Peacock AJ (2014). Right ventricle and high altitude. The Right Heart.

[14] Naeije R, Dedobbeleer C (2013). Pulmonary hypertension and the right ventricle in hypoxia. Exp Physiol.

[15] Rudski LG, Lai WW, Afilalo J, Hua L, Handschumacher MD, Chandrasekaran K, Solomon SD, Louie EK, Schiller NB (2010). Guidelines for the echocardiographic assessment of the right heart in adults: a report from the American society of echocardiography endorsed by the European association of echocardiography, a registered branch of the European society of cardiology, and the Canadian society of echocardiography. J Am Soc Echocardiogr.

[16] Seccombe LM, Chow V, Zhao W, Lau EM, Rogers PG, Ng AC, Veitch EM, Peters MJ, Kritharides L (2017). Right heart function during simulated altitude in patients with pulmonary arterial hypertension. Open Heart.

[17] Huez S, Retailleau K, Unger P, Pavelescu A, Vachiéry JL, Derumeaux G, Naeije R (2005). Right and left ventricular adaptation to hypoxia: a tissue doppler imaging study. Am J Physiol Heart Circ Physiol.

[18] Oknińska M, Zajda K, Zambrowska Z, Grzanka M, Paterek A, Mackiewicz U, Szczylik C, Kurzyna M, Piekiełko-Witkowska A, Torbicki A, Kieda C, Mączewski M (2024). Role of oxygen starvation in right ventricular decompensation and failure in pulmonary arterial hypertension. JACC Heart Fail.

[19] Lyon RC, Zanella F, Omens JH, Sheikh F (2015). Mechanotransduction in cardiac hypertrophy and failure. Circ Res.

[20] Gautel M (2011). The sarcomeric cytoskeleton: who picks up the strain?. Curr Opin Cell Biol.

[21] Johnson J, Yang Y, Bian Z, Schena G, Li Y, Zhang X, Eaton DM, Gross P, Angheloiu A, Shaik A, Foster M, Berretta R, Kubo H, Mohsin S, Tian Y, Houser SR (2023). Systemic hypoxemia induces cardiomyocyte hypertrophy and right ventricular specific induction of proliferation. Circ Res.

[22] Doutreleau S, Ulliel-Roche M, Hancco I, Bailly S, Oberholzer L, Robach P, Brugniaux JV, Pichon A, Stauffer E, Perger E, Parati G, Verges S (2022). Cardiac remodelling in the highest city in the world: effects of altitude and chronic mountain sickness. Eur J Prev Cardiol.

[23] Netzer NC, Strohl KP, Högel J, Gatterer H, Schilz R (2017). Right ventricle dimensions and function in response to acute hypoxia in healthy human subjects. Acta Physiol (Oxf).

[24] Pezzuto B, Forton K, Badagliacca R, Motoji Y, Faoro V, Naeije R (2018). Right ventricular dyssynchrony during hypoxic breathing but not during exercise in healthy subjects: a speckle tracking echocardiography study. Exp Physiol.

[25] Ewalts M, Dawkins T, Boulet LM, Thijssen D, Stembridge M (2021). The influence of increased venous return on right ventricular dyssynchrony during acute and sustained hypoxaemia. Exp Physiol.

[26] Alexander JK, Hartley LH, Modelski M, Grover RF (1967). Reduction of stroke volume during exercise in man following ascent to 3,100 m altitude. J Appl Physiol.

[27] Noakes TD (2000). Physiological models to understand exercise fatigue and the adaptations that predict or enhance athletic performance. Scand J Med Sci Sports.

[28] Welsh RC, Warburton DE, Humen DP, Taylor DA, McGavock J, Haykowsky MJ (2005). Prolonged strenuous exercise alters the cardiovascular response to dobutamine stimulation in male athletes. J Physiol.

[29] Larsen KO, Sjaastad I, Svindland A, Krobert KA, Skjønsberg OH, Christensen G (2006). Alveolar hypoxia induces left ventricular diastolic dysfunction and reduces phosphorylation of phospholamban in mice. Am J Physiol Heart Circ Physiol.

[30] Kjaergaard J, Snyder EM, Hassager C, Olson TP, Oh JK, Johnson BD, Frantz RP (2007). Right ventricular function with hypoxic exercise: effects of sildenafil. Eur J Appl Physiol.

[31] Stembridge M, Ainslie PN, Hughes MG, Stöhr EJ, Cotter JD, Nio AQ, Shave R (2014). Ventricular structure, function, and mechanics at high altitude: chronic remodeling in Sherpa vs. short-term lowlander adaptation. J Appl Physiol (1985).

[32] Forbes LM, Bull TM, Lahm T, Lawley JS, Hunter K, Levine BD, Lovering A, Roach RC, Subudhi AW, Cornwell WK (2023). Right ventricular response to acute hypoxia among healthy humans. Am J Respir Crit Care Med.

[33] Forbes LM, Bull TM, Lahm T, Sisson T, O’Gean K, Lawley JS, Hunter K, Levine BD, Lovering A, Roach RC, Subudhi AW, Cornwell WK (2024). Right ventricular performance during acute hypoxic exercise. J Physiol.

[34] Groves BM, Reeves JT, Sutton JR, Wagner PD, Cymerman A, Malconian MK, Rock PB, Young PM, Houston CS (1987). Operation Everest II: elevated high-altitude pulmonary resistance unresponsive to oxygen. J Appl Physiol (1985).

[35] Maufrais C, Rupp T, Bouzat P, Estève F, Nottin S, Walther G, Verges S (2019). Medex 2015: The key role of cardiac mechanics to maintain biventricular function at high altitude. Exp Physiol.

[36] Cornwell WK, Tran T, Cerbin L, Coe G, Muralidhar A, Hunter K, Altman N, Ambardekar AV, Tompkins C, Zipse M, Schulte M, O’Gean K, Ostertag M, Hoffman J, Pal JD, Lawley JS, Levine BD, Wolfel E, Kohrt WM, Buttrick P (2020). New insights into resting and exertional right ventricular performance in the healthy heart through real-time pressure-volume analysis. J Physiol.

[37] Brimioulle S, Wauthy P, Ewalenko P, Rondelet B, Vermeulen F, Kerbaul F, Naeije R (2003). Single-beat estimation of right ventricular end-systolic pressure-volume relationship. Am J Physiol Heart Circ Physiol.

[38] Frøbert O, Moesgaard J, Toft E, Poulsen SH, Søgaard P (2004). Influence of oxygen tension on myocardial performance. Evaluation by tissue Doppler imaging. Cardiovasc Ultrasound.

[39] Hanaoka M, Kogashi K, Droma Y, Urushihata K, Kubo K (2011). Myocardial performance index in subjects susceptible to high-altitude pulmonary edema. Intern Med.

[40] Reichenberger F, Kohstall MG, Seeger T, Olschewski H, Grimminger F, Seeger W, Ghofrani HA (2007). Effect of sildenafil on hypoxia-induced changes in pulmonary circulation and right ventricular function. Respir Physiol Neurobiol.

[41] Pagé M, Sauvé C, Serri K, Pagé P, Yin Y, Schampaert E (2013). Echocardiographic assessment of cardiac performance in response to high altitude and development of subclinical pulmonary edema in healthy climbers. Can J Cardiol.

[42] Tucker CE, James WE, Berry MA, Johnstone CJ, Grover RF (1976). Depressed myocardial function in the goat at high altitude. J Appl Physiol.

[43] Silverman HS, Wei S, Haigney MC, Ocampo CJ, Stern MD (1997). Myocyte adaptation to chronic hypoxia and development of tolerance to subsequent acute severe hypoxia. Circ Res.

[44] McConnell MV, Solomon SD, Rayan ME, Come PC, Goldhaber SZ, Lee RT (1996). Regional right ventricular dysfunction detected by echocardiography in acute pulmonary embolism. Am J Cardiol.

[45] Boehme J, Le Moan N, Kameny RJ, Loucks A, Johengen MJ, Lesneski AL, Gong W, Goudy BD, Davis T, Tanaka K, Davis A, He Y, Long-Boyle J, Ivaturi V, Gobburu JVS, Winger JA, Cary SP, Datar SA, Fineman JR, Krtolica A, Maltepe E (2018). Preservation of myocardial contractility during acute hypoxia with OMX-CV, a novel oxygen delivery biotherapeutic. PLoS Biol.

[46] He C, Liu C, Yu S, Yang J, Ding X, Bian S, Zhang J, Yu J, Tan H, Jin J, Hu M, Wu G, Zhang C, Rao R, Huang L (2021). Atrial performance in healthy subjects following high altitude exposure at 4100 m: 2D speckle-tracking strain analysis. Int J Cardiovasc Imaging.

[47] Dandel M, Hetzer R (2016). Echocardiographic assessment of the right ventricle: Impact of the distinctly load dependency of its size, geometry and performance. Int J Cardiol.

[48] Mason NP, Barry PW, Pollard AJ, Collier DJ, Taub NA, Miller MR, Milledge JS (2000). Serial changes in spirometry during an ascent to 5,300 m in the Nepalese Himalayas. High Alt Med Biol.

[49] Wilkins MR, Ghofrani HA, Weissmann N, Aldashev A, Zhao L (2015). Pathophysiology and treatment of high-altitude pulmonary vascular disease. Circulation.

[50] Hackett PH, Roach RC (2001). High-altitude illness. N Engl J Med.

[51] Imray C, Wright A, Subudhi A, Roach R (2010). Acute mountain sickness: pathophysiology, prevention, and treatment. Prog Cardiovasc Dis.

[52] Allemann Y, Rotter M, Hutter D, Lipp E, Sartori C, Scherrer U, Seiler C (2004). Impact of acute hypoxic pulmonary hypertension on LV diastolic function in healthy mountaineers at high altitude. Am J Physiol Heart Circ Physiol.

[53] Huez S, Faoro V, Guénard H, Martinot JB, Naeije R (2009). Echocardiographic and tissue doppler imaging of cardiac adaptation to high altitude in native highlanders versus acclimatized lowlanders. Am J Cardiol.

[54] Maufrais C, Rupp T, Bouzat P, Doucende G, Verges S, Nottin S, Walther G (2017). Heart mechanics at high altitude: 6 days on the top of Europe. Eur Heart J Cardiovasc Imaging.

[55] Gillebert TC, Leite-Moreira AF, De Hert SG (1997). Relaxation-systolic pressure relation. A load-independent assessment of left ventricular contractility. Circulation.

[56] Naeije R (2018). Cœur droit et altitude. Rev Mal Respir.

[57] Pugh LGCE (1964). Cardiac output in muscular exercise at 5,800 m (19,000 ft). J Appl Physiol.

[58] Hartley LH, Alexander JK, Modelski M, Grover RF (1967). Subnormal cardiac output at rest and during exercise in residents at 3,100 m altitude. J Appl Physiol.

[59] Reeves JT, Groves BM, Sutton JR, Wagner PD, Cymerman A, Malconian MK, Rock PB, Young PM, Houston CS (1987). Operation Everest II: preservation of cardiac function at extreme altitude. J Appl Physiol (1985).

[60] Hansen J, Sander M (2003). Sympathetic neural overactivity in healthy humans after prolonged exposure to hypobaric hypoxia. J Physiol.

[61] Parati G, Bilo G, Faini A, Bilo B, Revera M, Giuliano A, Lombardi C, Caldara G, Gregorini F, Styczkiewicz K, Zambon A, Piperno A, Modesti PA, Agostoni P, Mancia G (2014). Changes in 24 h ambulatory blood pressure and effects of angiotensin II receptor blockade during acute and prolonged high-altitude exposure: a randomized clinical trial. Eur Heart J.

[62] Ruiz L, Peñaloza D (1977). Altitude and hypertension. Mayo Clin Proc.

[63] Klausen K (1966). Cardiac output in man in rest and work during and after acclimatization to 3,800 m. J Appl Physiol.

[64] Vogel JA, Harris CW (1967). Cardiopulmonary responses of resting man during early exposure to high altitude. J Appl Physiol.

[65] Bogaard HJ, Hopkins SR, Yamaya Y, Niizeki K, Ziegler MG, Wagner PD (2002). Role of the autonomic nervous system in the reduced maximal cardiac output at altitude. J Appl Physiol (1985).

[66] Cunningham WL, Becker EJ, Kreuzer F (1965). Catecholamines in plasma and urine at high altitude. J Appl Physiol.

[67] Mazzeo RS, Brooks GA, Butterfield GE, Podolin DA, Wolfel EE, Reeves JT (1995). Acclimatization to high altitude increase muscle sympathetic activity both at rest and during exercise. Am J Physiol.

[68] Stenberg J, Ekblom B, Messin R (1966). Hemodynamic response to work at simulated altitude, 4,000 m. J Appl Physiol.

[69] Suarez J, Alexander JK, Houston CS (1987). Enhanced left ventricular systolic performance at high altitude during Operation Everest II. Am J Cardiol.

[70] Boussuges A, Molenat F, Burnet H, Cauchy E, Gardette B, Sainty JM, Jammes Y, Richalet JP (2000). Operation Everest III (Comex ‘97): modifications of cardiac function secondary to altitude-induced hypoxia. An echocardiographic and doppler study. Am J Respir Crit Care Med.

[71] Alexander JK, Grover RF (1983). Mechanism of reduced cardiac stroke volume at high altitude. Clin Cardiol.

[72] Stembridge M, Ainslie PN, Hughes MG, Stöhr EJ, Cotter JD, Tymko MM, Day TA, Bakker A, Shave R (2015). Impaired myocardial function does not explain reduced left ventricular filling and stroke volume at rest or during exercise at high altitude. J Appl Physiol (1985).

[73] Grover RF, Reeves JT, Maher JT, McCullough RE, Cruz JC, Denniston JC, Cymerman A (1976). Maintained stroke volume but impaired arterial oxygenation in man at high altitude with supplemental CO2. Circ Res.

[74] Grover RF, Reeves JT, Grover EB, Leathers JE (1967). Muscular exercise in young men native to 3,100 m altitude. J Appl Physiol.

[75] Sylvester JT, Shimoda LA, Aaronson PI, Ward JP (2012). Hypoxic pulmonary vasoconstriction. Physiol Rev.

[76] Penaloza D, Arias-Stella J (2007). The heart and pulmonary circulation at high altitudes: healthy highlanders and chronic mountain sickness. Circulation.

[77] McIntyre KM, Sasahara AA (1971). The hemodynamic response to pulmonary embolism in patients without prior cardiopulmonary disease. Am J Cardiol.

[78] MacNee W (1994). Pathophysiology of cor pulmonale in chronic obstructive pulmonary disease. Part two. Am J Respir Crit Care Med.

[79] Peñaloza D, Sime F (1971). Chronic cor pulmonale due to loss of altitude acclimatization (chronic mountain sickness). Am J Med.

[80] Brito J, Siques P, López R, Romero R, León-Velarde F, Flores K, Lüneburg N, Hannemann J, Böger RH (2018). Long-term intermittent work at high altitude: right heart functional and morphological status and associated cardiometabolic factors. Front Physiol.

[81] Archer SL, Dunham-Snary KJ, Bentley R, Alizadeh E, Weir EK (2024). Hypoxic pulmonary vasoconstriction: an important component of the homeostatic oxygen sensing system. Physiol Res.

[82] McLoughlin P, Ward JP (2013). Hypoxic pulmonary hypertension; the load on the right ventricle. Introduction. Exp Physiol.

[83] West JB (2012). High-altitude medicine. Am J Respir Crit Care Med.

[84] Soria R, Egger M, Scherrer U, Bender N, Rimoldi SF (2016). Pulmonary artery pressure and arterial oxygen saturation in people living at high or low altitude: systematic review and meta-analysis. J Appl Physiol (1985).

[85] Tian J, Liu C, Yang Y, Yu S, Yang J, Zhang J, Ding X, Zhang C, Rao R, Zhao X, Huang L (2020). Effects of baseline heart rate at sea level on cardiac responses to high-altitude exposure. Int J Cardiovasc Imaging.

[86] Groepenhoff H, Overbeek MJ, Mulè M, van der Plas M, Argiento P, Villafuerte FC, Beloka S, Faoro V, Macarlupu JL, Guenard H, de Bisschop C, Martinot JB, Vanderpool R, Penaloza D, Naeije R (2012). Exercise pathophysiology in patients with chronic mountain sickness exercise in chronic mountain sickness. Chest.

[87] Banchero N, Sime F, Peñaloza D, Cruz J, Gamboa R, Marticorena E (1966). Pulmonary pressure, cardiac output, and arterial oxygen saturation during exercise at high altitude and at sea level. Circulation.

[88] Naeije R (2011). Pro: Hypoxic pulmonary vasoconstriction is a limiting factor of exercise at high altitude. High Alt Med Biol.

[89] Simonneau G, Montani D, Celermajer DS, Denton CP, Gatzoulis MA, Krowka M, Williams PG, Souza R (2019). Haemodynamic definitions and updated clinical classification of pulmonary hypertension. Eur Respir J.

[90] León-Velarde F, Maggiorini M, Reeves JT, Aldashev A, Asmus I, Bernardi L, Ge RL, Hackett P, Kobayashi T, Moore LG, Penaloza D, Richalet JP, Roach R, Wu T, Vargas E, Zubieta-Castillo G, Zubieta-Calleja G (2005). Consensus statement on chronic and subacute high altitude diseases. High Alt Med Biol.

[91] Naeije R (2015). Assessment of right ventricular function in pulmonary hypertension. Curr Hypertens Rep.

[92] Talbot NP, Balanos GM, Dorrington KL, Robbins PA (2005). Two temporal components within the human pulmonary vascular response to approximately 2 h of isocapnic hypoxia. J Appl Physiol (1985).

[93] Dunham-Snary KJ, Wu D, Sykes EA, Thakrar A, Parlow LRG, Mewburn JD, Parlow JL, Archer SL (2017). Hypoxic pulmonary vasoconstriction: from molecular mechanisms to medicine. Chest.

[94] Duplain H, Vollenweider L, Delabays A, Nicod P, Bärtsch P, Scherrer U (1999). Augmented sympathetic activation during short-term hypoxia and high-altitude exposure in subjects susceptible to high-altitude pulmonary edema. Circulation.

[95] Villafuerte FC, Simonson TS, Bermudez D, León-Velarde F (2022). High-altitude erythrocytosis: mechanisms of adaptive and maladaptive responses. Physiology (Bethesda).

[96] Bačáková L, Sedlář A, Musílková J, Eckhardt A, Žaloudíková M, Kolář F, Maxová H (2024). Mechanisms controlling the behavior of vascular smooth muscle cells in hypoxic pulmonary hypertension. Physiol Res.

[97] Vogel JH, Weaver WF, Rose RL, Blount SG, Grover RF (1962). Pulmonary hypertension on exertion in normal man living at 10,150 feet (Leadville, Colorado). Med Thorac.

[98] Weitzenblum E (1994). The pulmonary circulation and the heart in chronic lung disease. Monaldi Arch Chest Dis.

[99] Kholdani C, Fares WH, Mohsenin V (2015). Pulmonary hypertension in obstructive sleep apnea: is it clinically significant? A critical analysis of the association and pathophysiology. Pulm Circ.

